# Comparison of the Effects of Sevoflurane and Isoflurane During Liver Transplant Surgery on the Short-Term Cardiac, Hepatic, and Renal Outcome: A Randomized Clinical Trial

**DOI:** 10.5812/ijpr-166290

**Published:** 2026-02-11

**Authors:** Mohammadreza Moshari, Sadaf Tahery, Mastaneh Dahi Taleghani, Shide Dabir, Maryam Vosoughian, Soudeh Tabashi, Mohsen Ariannik, Firoozeh Madadi

**Affiliations:** 1 Department of Anesthesiology, Anesthesiology Research Center, School of Medicine, Shahid Beheshti University of Medical Sciences, Tehran, Iran; 2 Department of Anesthesiology, School of Medicine, Shahid Beheshti University of Medical Sciences, Tehran, Iran; 3 Anesthesiology Research Center, Shahid Beheshti University of Medical Sciences, Tehran, Iran; 4 Department of Anesthesiology, Ayatollah Taleghani Hospital, School of Medicine, Shahid Beheshti University of Medical Sciences, Tehran, Iran

**Keywords:** Sevoflurane, Isoflurane, Liver Transplant Surgery, Postoperative Complications, Ischemia-Reperfusion Injury

## Abstract

**Background:**

Liver transplantation is frequently complicated by ischemia-reperfusion injury (IRI), which may impair hepatic, renal, and cardiac function. Volatile anesthetics such as isoflurane and sevoflurane are believed to mitigate this injury.

**Objectives:**

This study aimed to compare their effects on short-term organ outcomes in deceased donor liver transplant recipients.

**Methods:**

In this study, 70 liver transplantation candidates at Taleghani Hospital in Tehran were enrolled after obtaining informed consent, and various variables were assessed before, during, and at two intervals immediately after surgery and one week post-operation. Patients were randomly allocated to receive either isoflurane or sevoflurane for anesthesia maintenance using the sealed opaque envelope technique for allocation concealment. Randomization was performed by a study nurse not involved in patient care using computer-generated random numbers. The primary outcome was defined as the postoperative peak serum alanine aminotransferase (ALT) level. Secondary outcomes included peak aspartate aminotransferase (AST), total bilirubin, creatinine, troponin I, C-reactive protein (CRP), intraoperative blood product requirements (packed red blood cells and fresh frozen plasma), hemodynamic parameters, and urine output.

**Results:**

Baseline characteristics were comparable between groups. No significant differences were found in intraoperative hemodynamics or postoperative laboratory values of liver and renal function (P > 0.05). Postoperative liver enzyme levels increased in both groups following reperfusion, consistent with IRI. However, no statistically significant differences were observed between the isoflurane and sevoflurane groups in peak serum ALT levels, measured 6 hours after reperfusion and on postoperative day 7 (P > 0.05 for all comparisons). Similarly, AST levels did not differ significantly between groups at any postoperative time point. Renal and cardiac biomarkers, including creatinine and troponin I, were also comparable between groups. In contrast, patients receiving sevoflurane required significantly higher volumes of packed red blood cells and fresh frozen plasma intraoperatively compared with the isoflurane group (P < 0.05).

**Conclusions:**

The results obtained in this study showed that the use of isoflurane and sevoflurane did not have a significant difference in the severity of ischemic reperfusion injury caused after liver transplantation surgery on the liver, kidney, and heart; also, in this study, the functional conditions of these organs during and after surgery were evaluated, and by examining at different time intervals, these two inhalation anesthetics did not have a different effect on the short-term outcome of patients after receiving a liver transplant.

## 1. Background

Liver transplantation is the definitive treatment for end-stage liver disease and has greatly improved in recent decades due to advances in surgical techniques, organ preservation, and immunosuppressive therapy (1, 2). Despite these advances, ischemia-reperfusion injury (IRI) remains a significant challenge, contributing to graft dysfunction and failure post-transplantation (3-5). Ischemia-reperfusion injury occurs when blood supply returns to the liver after a period of ischemia, triggering a cascade of inflammatory responses, oxidative stress, and cellular damage that extend beyond the liver to affect other organs, including the heart and kidneys (6-9).

The pathophysiology of IRI involves complex molecular mechanisms. Oxygen deprivation during ischemia leads to anaerobic glycolysis, acidosis, and cellular ion imbalances. Upon reperfusion, the sudden restoration of oxygen supply causes overproduction of reactive oxygen species (ROS), primarily by Kupffer cells and neutrophils. Reactive oxygen species initiate lipid peroxidation, mitochondrial damage, and activation of pro-inflammatory pathways, including cytokine release, inflammasome activation, and pyroptosis (6, 10-13). This cascade not only worsens hepatic injury but can propagate systemic inflammation and multiorgan dysfunction. Factors such as hepatic steatosis further increase susceptibility to IRI due to impaired microcirculation and reduced antioxidant capacity (14-16).

Strategies to mitigate IRI include optimal perfusion techniques, preconditioning, and pharmacologic interventions (17, 18). Among these, the choice of anesthetic agents during transplantation has attracted attention due to their immunomodulatory and organ-protective effects. Inhalational anesthetics such as isoflurane and sevoflurane have shown potential in attenuating IRI. Isoflurane has demonstrated anti-inflammatory effects and the ability to modulate apoptotic pathways via NF-κB and AKT signaling (7, 19). Sevoflurane, on the other hand, may confer hepatoprotective benefits by reducing oxidative stress, regulating the Nrf2/HO-1 pathway, and limiting cellular apoptosis (20-24).

However, clinical findings comparing these two agents have been mixed. Some studies report reduced hepatic injury and better postoperative outcomes with sevoflurane (22, 25), while others find no significant difference or even favor isoflurane in terms of intraoperative hemodynamic stability or bleeding (19, 26, 27). Additionally, potential side effects such as nephrotoxicity linked to fluoride metabolites from sevoflurane metabolism have raised concerns (20, 21, 23).

## 2. Objectives

Given this background, the optimal choice of volatile anesthetic in liver transplant surgery remains controversial. This study aims to directly compare the effects of isoflurane and sevoflurane on hepatic, renal, and cardiac outcomes during and after deceased donor liver transplantation. By analyzing intraoperative parameters, biochemical markers, and postoperative complications, the study seeks to determine whether either anesthetic provides superior organ protection. The goal is to provide evidence-based guidance for anesthetic selection in deceased donor liver transplant patients, potentially contributing to improved short-term outcomes and better graft preservation.

## 3. Methods

### 3.1. Trial Design and Setting

We conducted a single-center, prospective, parallel-group, randomized clinical trial at the Organ Transplant Surgery Department of Ayatollah Taleghani Hospital (Tehran, Iran) between December 2024 and April 2025. The study protocol was approved by the Ethics Committee of Shahid Beheshti University of Medical Sciences (IR.SBMU.MSP.REC.1403.160) and registered at the Iranian Registry of Clinical Trials before patient enrollment (IRCT20240909062992N1, registration date: 2024-10-22). All participants provided written informed consent before enrollment.

### 3.2. Participants

Adult patients (≥ 18 years) listed for orthotopic liver transplantation from deceased donor were screened. Inclusion criteria: Eligibility for liver transplant; ability to consent. Exclusion criteria: Emergency surgery; cardiopulmonary resuscitation during anesthesia; failure to extubate within 24 h; massive intraoperative hemorrhage (> 1 h anhepatic phase or excessive bleeding); death within 7 days.

### 3.3. Randomization and Blinding

After eligibility confirmation and consent, 70 patients were randomized 1:1 to receive isoflurane or sevoflurane. A computer-generated random sequence (block size = 4) was prepared by an independent statistician. Allocation concealment was maintained using sequentially numbered, opaque, sealed envelopes. An anesthesia technician, not involved in data collection, opened the envelope immediately before induction. Care providers and outcome assessors remained blinded to group assignment; anesthesiologists could not be blinded due to the nature of the intervention but did not participate in data collection.

### 3.4. Interventions

Anesthetic induction (both groups): Midazolam 0.05 mg/kg, fentanyl 2 - 3 µg/kg, propofol 1.5 - 2 mg/kg, lidocaine 1 mg/kg, cisatracurium 0.15 mg/kg. Maintenance: Isoflurane group: Isoflurane 1.0 - 1.5 MAC Sevoflurane group: Sevoflurane 1.7 - 2.6 MAC Bispectral Index (BIS) was kept between 40 and 60. Intraoperative monitoring included invasive arterial pressure (LiDCO device), central venous pressure, heart rate, urine output, fluid balance, and inotrope use.

### 3.5. Outcome Measures

Primary outcome: Laboratory markers [alanine aminotransferase (ALT), aspartate aminotransferase (AST), bilirubin, creatinine, troponin, C-reactive protein (CRP)] measured preoperatively, 6 hours post-reperfusion, and on postoperative day 7. Secondary outcomes: Hemodynamic parameters (cardiac output, systemic vascular resistance) and urine output measured at preanhepatic, anhepatic, and reperfusion phases · Inotrope requirement (yes/no) during each phase. Intraoperative transfusion volume (packed red blood cells and fresh frozen plasma). Adverse events (bleeding, arrhythmias, acute kidney injury) were recorded throughout hospitalization.

### 3.6. Sample Size

The sample size was calculated based on the primary outcome, defined as the postoperative peak serum ALT level as a surrogate marker of hepatic ischemia–reperfusion injury. Based on previously published studies in liver transplantation and pilot institutional data, a clinically meaningful difference of 250 U/L in peak postoperative ALT between the isoflurane and sevoflurane groups was assumed, with an estimated standard deviation of 300 U/L. Using a two-sided independent samples *t*-test, an alpha level of 0.05, and a statistical power of 80%, a minimum of 32 patients per group was required to detect this difference. To account for potential dropouts, protocol violations, and early postoperative exclusions inherent to liver transplantation studies, the target enrollment was increased by approximately 10%, resulting in a planned sample size of 35 patients per group (70 patients in total).

### 3.7. Statistical Analysis

Data were analyzed using SPSS v.24. Continuous variables were tested for normality (Kolmogorov-Smirnov) and compared using Student’s *t*-test or Mann-Whitney U test as appropriate. Categorical variables were compared using χ² test or Fisher’s exact test. Repeated measures (laboratory values across time points) were analyzed by two-way ANOVA with Bonferroni correction. A P-value < 0.05 was considered statistically significant. Analyses followed the intention-to-treat principle. Data are presented as mean ± SD or median (interquartile range) as appropriate. P-values are unadjusted; no correction for multiple comparisons was applied because all biochemical outcomes except transfusion requirements were considered exploratory/secondary.

## 4. Results

**Figure 1.**
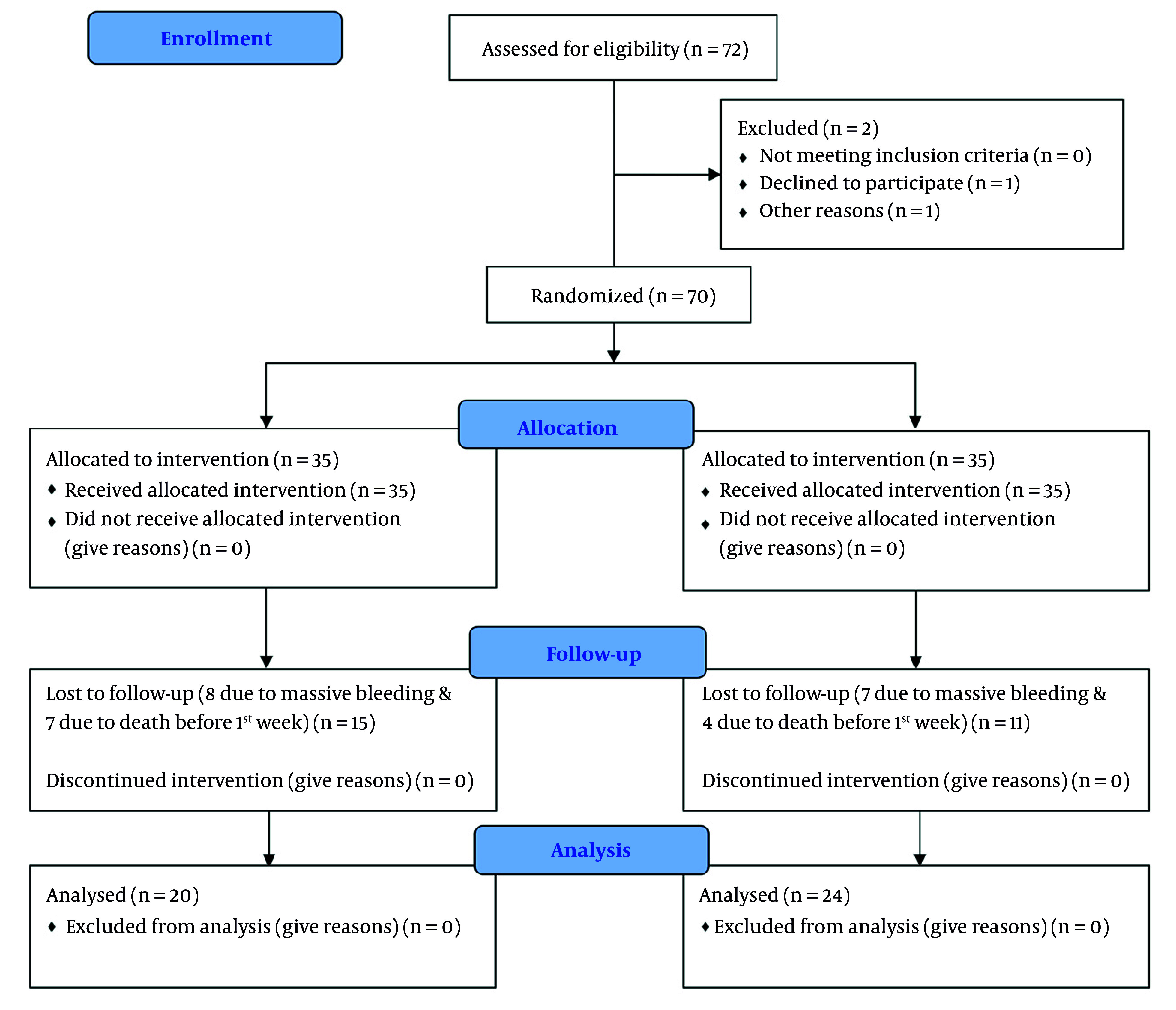
Flow diagram of patient enrollment in the study

A total of 70 patients were initially enrolled and randomized into two groups. Following the exclusion of patients based on predefined criteria, 44 participants completed the study protocol — 20 in the isoflurane group and 24 in the sevoflurane group (Figure 1). Baseline demographic and clinical characteristics, including age, sex, MELD score, and etiology of liver disease, were similar between the two groups, with no statistically significant differences (Table 1). 

**Table 1.** Demographic Characteristics of the Patients ^a^

Variables	Isoflurane	Sevoflurane	P-Value
**Gender**	10 males	11 males	0.402
**Age (y)**	44.35 ± 12.014	44.25 ± 13.829	0.980
**BMI (kg/m** ^**2**^ **)**	23.46 ± 3.182	24.93 ± 4.50	0.185
**MELD score**	23.55 ± 4.019	24.04 ± 6.597	0.763
**Donors age (y)**	38.15 ± 11.940	43.88 ± 14.128	0.159
**Organ weight (g)**	1373.50 ± 114.812	1402.71 ± 292.288	0.677

^a^ Values are expressed as mean ± SD.

Regarding the primary outcomes, there were no significant differences in postoperative laboratory markers between the groups. Serum levels of ALT, AST, total bilirubin, creatinine, troponin, and CRP were measured preoperatively, 6 hours after reperfusion, and on postoperative day 7. While both groups demonstrated postoperative elevations consistent with the surgical context, no statistically significant differences were observed at any time point for any of the measured biochemical parameters between the two groups (P > 0.05 for all comparisons) (Table 2). 

**Table 2.** Laboratory Data of Two Groups of Patients in 3 Different Time Points ^a^

Laboratory Data/Sampling Time	Isoflurane	Sevoflurane	P-Value
**Cr (mg/dL)**			
Preoperative	1.10 ± 0.79	1.08 ± 0.81	0.947
6 hrs postop	1.13 ± 0.71	1.09 ± 0.73	0.865
7 d postop	1.12 ± 0.55	1.01 ± 0.50	0.486
**ALT (U/L)**			
Preoperative	145.80 ± 386.72	232.46 ± 397.61	0.470
6 hrs postop	458.20 ± 815.91	696.38 ± 864.17	0.356
7 d postop	221.25 ± 362.13	244.29 ± 309.80	0.821
**AST (U/L)**			
Preoperative	287.95 ± 995.57	309.75 ± 484.05	0.925
6 hrs postop	511.40 ± 910.26	698.33 ± 635.82	9.428
7 d postop	161.05 ± 359.104	944.54 ± 3919.79	0.379
**Total bilirubin (mg/dL)**			
Preoperative	9.86 ± 26.26.18	9.10 ± 10.74	0.898
6 hrs postop	10.25 ± 27.30	34.28 ± 124.61	0.403
7 d postop	3.33 ± 3.24	4.35 ± 5.10	0.442
**Troponin (ng/mL)**			
Preoperative	0.18 ± 0.60	0.01 ± 0.01	0.228
6 hrs postop	0.05 ± 0.06	0.10 ± 0.19	0.237
7 d postop	0.09 ± 0.10	0.09 ± 0.12	0.965
**CRP (mg/L)**			
Preoperative	25.13 ± 20.52	23.90 ± 29.17	0.875
6 hrs postop	27.38 ± 18.27	40.77 ± 40.97	0.159
7 d postop	28.94 ± 23.39	37.89 ± 39.77	0.378

Abbreviations: Cr, creatinine; ALT, alanine aminotransferase; AST, aspartate aminotransferase; CRP, C-reactive protein.

^a^ Values are expressed as mean ± SD.

In terms of secondary outcomes, intraoperative hemodynamic variables, including cardiac output, systemic vascular resistance, and urine output, were recorded during the preanhepatic, anhepatic, and reperfusion phases. These parameters did not differ significantly between the isoflurane and sevoflurane groups across any phase of surgery (P > 0.05). Additionally, the requirement for inotropic support during each phase was comparable between groups, with no statistically significant differences during the preanhepatic (P = 0.87), anhepatic (P = 0.66), or reperfusion (P = 0.47) periods (Table 3). 

**Table 3.** Intraoperative Variables of Patients ^a^

Parameters/Surgery Phases	Isoflurane	Sevoflurane	P-Value
**Urine output (mL)**			
Preanhepatic	968.00 ± 707.595	689.58 ± 556.257	0.151
Anhepatic	381.00 ± 454.346	281.25 ± 343.832	0.412
Reperfusion	935.00 ± 568.493	775.00 ± 482.070	0.318
**Inotrope (norepinephrine)**			
Preanhepatic	35.0	50.0	0.244
Anhepatic	85.0	91.7	0.411
Reperfusion	55.0	79.2	0.082
**Cardiac output (L/min)**			
Preanhepatic	6.180 ± 1.788	6.10 ± 2.147	0.895
Anhepatic	5.035 ± 1.756	5.60 ± 2.512	0.395
Reperfusion	8.455 ± 2.863	8.44 ± 3.195	0.989
**Systemic vascular resistance (dyne × sec/cm** ^**5**^ **)**			
Preanhepatic	1012.10 ± 400.20	907.00 ± 335.27	0.348
Anhepatic	1137.25 ± 625.03	1011.00 ± 511.19	0.465
Reperfusion	760.30 ± 304.369	782.75 ± 312.05	0.811
**Cardiac Index (L/min/m** ^**2**^ **)**			
Preanhepatic	3.915 ± 1.030	4.10 ± 2.28	0.740
Anhepatic	3.050 ± 0.915	3.86 ± 1.83	0.065
Reperfusion	4.935 ± 1.588	4.67 ± 1.65	0.593
**Bicarbonate (mEq)**			
Preanhepatic	52.00 ± 23.02	69.79 ± 45.43	0.102
Anhepatic	81.75 ± 52.84	108.33 ± 54.50	0.110
Reperfusion	44.75 ± 26.13	62.50 ± 44.84	0.126

^a^ Values are expressed as mean ± SD.

Notably, a significant difference was observed in intraoperative transfusion requirements. Patients in the sevoflurane group required a greater volume of packed red blood cells (2.88 ± 1.73 units) compared to those in the isoflurane group (1.75 ± 1.71 units; P = 0.037). Similarly, the volume of fresh frozen plasma administered was higher in the sevoflurane group (2.42 ± 1.69 units vs. 1.25 ± 1.68 units; P = 0.028), indicating a greater transfusion burden associated with sevoflurane (Table 4). 

**Table 4.** Intraoperative Conditions of Patients in Terms of Bleeding and Received Fluids and Products ^a^

Variables	Isoflurane	Sevoflurane	P-Value
**Bleeding rate**	920.00 ± 501.157	1228.33 ± 666.664	0.096
**Received pack cell**	1.75 ± 1.713	2.88 ± 1.727	0.037
**Received FFP**	1.25 ± 1.682	2.42 ± 1.692	0.028
**Amount of crystalloids**	4225.00 ± 1129.450	4182.50 ± 1071.493	0.911
**Amount of albumin**	82.50 ± 81.556	66.67 ± 80.307	0.522

Abbreviation: FFP, fresh frozen plasma.

^a^ Values are expressed as mean ± SD.

As for adverse events, no significant differences were noted between the two groups. The incidence of postoperative bleeding, cardiac arrhythmias, and acute kidney injury was low and similar in both groups. No patients required reoperation or renal replacement therapy during the postoperative period, and no deaths were recorded within the early postoperative phase.

## 5. Discussion

This randomized clinical trial compared the effects of isoflurane and sevoflurane on short-term hepatic, renal, and cardiac outcomes in patients undergoing liver transplantation. The results demonstrated no significant differences between the two anesthetic agents in postoperative laboratory markers, intraoperative hemodynamics, or inotropic requirements. However, the use of sevoflurane was associated with a significantly higher intraoperative transfusion volume compared to isoflurane.

Our findings align with several previous studies suggesting that both isoflurane and sevoflurane can be safely used for anesthetic maintenance in liver transplantation, with comparable impacts on organ function (22, 27). The lack of difference in postoperative ALT, AST, bilirubin, creatinine, troponin, and CRP levels suggests that neither agent confers a clear advantage in attenuating IRI at the systemic level. This is consistent with the mechanistic similarities between the two agents, both of which have been shown to exert anti-inflammatory and antioxidant effects through modulation of pathways such as NF-κB, AKT, and Nrf2/HO-1 in preclinical studies (24, 28, 29).

Intraoperative hemodynamics, including cardiac output and systemic vascular resistance, were stable and comparable between the groups, supporting previous findings that both volatile anesthetics are hemodynamically acceptable in the setting of liver transplantation (27, 30, 31). Similarly, inotropic support requirements were not significantly different, suggesting no clear superiority of one agent over the other in cardiovascular performance during surgery.

Importantly, no major adverse events such as arrhythmias, acute kidney injury, or early graft failure occurred in either group, indicating that both anesthetics were well tolerated. While the observed difference in transfusion volume may have clinical implications — given the known risks associated with blood product administration — the short-term outcomes did not reveal any organ-specific complications linked to either anesthetic.

Interestingly, our study observed a significantly greater need for packed red blood cell and plasma transfusions in the sevoflurane group. Although the mechanisms underlying this difference are unclear, some reports have suggested that sevoflurane may interfere with coagulation or enhance fibrinolysis, potentially leading to increased bleeding (26, 32). However, these effects remain controversial and warrant further investigation in larger trials specifically designed to assess coagulation profiles.

The findings of this study can help us choose anesthetic drugs based on the coagulation status of each patient. For instance, Eyth et al. developed a model that can predict intraoperative blood transfusion (33). When an anesthesiologist identifies a patient with a higher risk of bleeding based on this model and similar models, they will choose an anesthetic drug with a lower risk of transfusion need, such as isoflurane, as explained in our study. However, this study has a small population. To integrate these findings into clinical practice, future studies will be necessary. Future studies on this area can be categorized into three main areas. First, the findings of this paper should be confirmed in larger studies. Second, more anesthetic drugs should be taken into consideration for such comparisons. Third, since blood transfusion is a determinant factor in liver transplant success (34), we may be able to integrate anesthesia drugs into risk score systems and predictive systems in liver transplant studies (35, 36).

Limitations Given the final sample size of 44 patients and the high variability typically seen in postoperative liver enzyme levels after liver transplantation, the study had limited statistical power to detect small-to-moderate differences in biochemical markers of organ injury or to formally demonstrate equivalence.

### 5.1. Conclusions

The results obtained in this study showed that the use of isoflurane and sevoflurane did not have a significant difference in the severity of ischemic reperfusion injury caused after liver transplantation surgery on the liver, kidney, and heart; also, in this study, the functional conditions of these organs during and after surgery were evaluated, and by examining at different time intervals, these two inhalation anesthetics did not have a different effect on the short-term outcome of patients after receiving a liver transplant.

## Data Availability

The dataset presented in the study is available on request from the corresponding author during submission or after publication.

## Clinical Trial Registration Code:

IRCT20240909062992N1
